# Pulmonary mucoepidermoid carcinoma in children: two case reports and a review of the literature

**DOI:** 10.3389/fped.2023.1232185

**Published:** 2023-09-12

**Authors:** Yuan Huang, Yong Fu, Jing Sun, Bin Xu, Lei Wu, Lan-fang Tang

**Affiliations:** ^1^ Department of Pulmonology, Children's Hospital, Zhejiang University School of Medicine, National Clinical Research Center for Child Health, Hangzhou, China; ^2^ Department of Endoscopy Center, Children's Hospital, Zhejiang University School of Medicine, National Clinical Research Center for Child Health, Hangzhou, China; ^3^ Department of Otorhinolaryngology-Head and Neck Surgery, Children's Hospital, Zhejiang University School of Medicine, National Clinical Research Center for Child Health, Hangzhou, China

**Keywords:** pulmonary mucoepidermoid carcinoma (PMEC), children, flexible bronchoscopy, atelectasis, surgery

## Abstract

Pulmonary mucoepidermoid carcinoma (PMEC) is a rare tumor, particularly in children, and its clinical manifestations vary. When the tumor is small, it may be asymptomatic; however, with larger tumors, patients may present with symptoms such as recurring pneumonia, atelectasis, persistent cough, chest pain, and even hemoptysis. PMEC appears as an exophytic intrabronchial mass. This study aims to report on the clinical manifestations, imaging findings, treatment approaches, and prognosis of two children diagnosed with PMEC at our hospital between January 2018 and December 2022. The age of onset for both children was 9 years, and the masses were located in the right upper lobe bronchi. Following surgical treatment, both patients showed a good prognosis. In addition, we conducted a comprehensive review of the relevant literature to enhance the overall understanding of PMEC.

## Introduction

Pulmonary mucoepidermoid carcinoma (PMEC) originates in the submucosal bronchial glands and is a rare malignant tumor. It constitutes 2.5%–7.3% and 0.1%–0.2% of endobronchial adenomas and primary lung cancers, respectively (1). The incidence of PMEC is extremely low in children, with 55 cases reported (1). From January 2018 to December 2022, we identified and analyzed two cases of PMEC that were diagnosed at our hospital. In this study, we emphasize the clinical features, treatment outcomes, and prognosis of these cases.

## Case presentation

### Case 1

**Figure 1:**
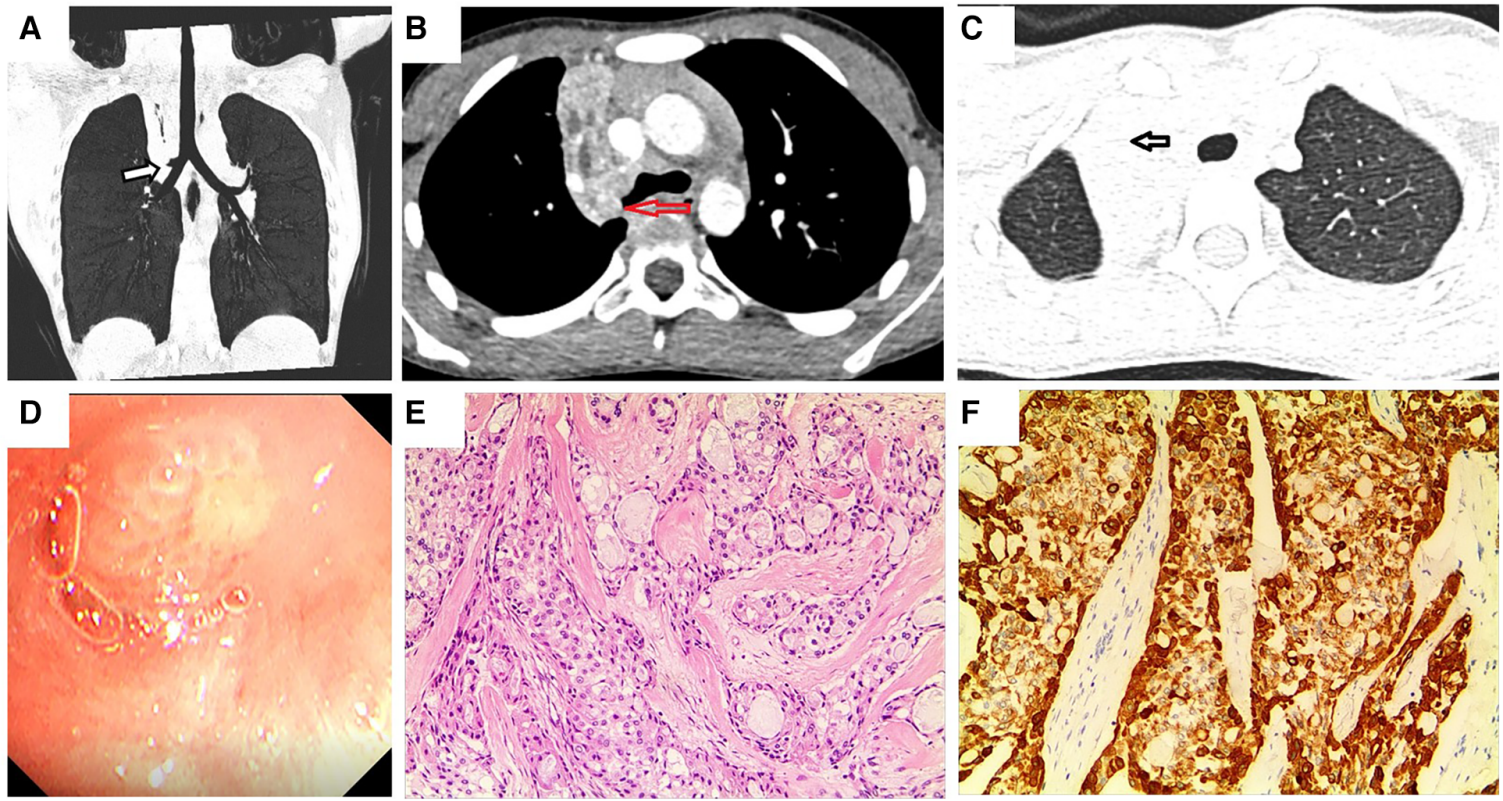
Chest enhanced CT, bronchoscopy, pathological image, and immunohistochemistry assay of case 1. (**A,B**) Contrast-enhanced CT images of the chest show a space-occupying lesion at the right upper lobe bronchial opening. Arrows indicate space-occupying lesions. (**C**) Arrow indicates right upper lobe atelectasis. (**D**) Visual depiction of flexible bronchoscopy, confirming the presence of a ball-shaped soft solid tumor originating from the right upper lobe bronchus. (**E**) Photomicrograph (HE × 100) shows the confirmed diagnosis of pulmonary mucoepidermoid carcinoma. (**F**) Photomicrograph shows positive immunohistochemical staining for CK (magnification × 100).

A previously healthy 9-year-old girl was admitted to the hospital with persistent cough and chest pain for over 10 days. Remarkably, she showed no current fever, hemoptysis, nausea, abdominal pain, vomiting, diarrhea, chest pain, palpitations, or weight loss. Moreover, her medical history was devoid of any surgeries, traumas, encounters with sick individuals, aspiration episodes, or exposure to infectious and/or hazardous substances. Furthermore, she had no family history of asthma, diabetes, immunodeficiency, malignancy, or tuberculosis. The patient had encountered a bout of upper right pneumonia more than a year before the current presentation. However, after the patient's respiratory symptoms improved, the chest radiograph was not reviewed, and no further tests were performed. On the fifth day of experiencing chest pain, the patient visited a local hospital. Subsequent chest CT showed atelectasis of the upper lobe of the right lung, and flexible bronchoscopy was recommended. Her parents referred her to our facility for further evaluation and management. Blood routine and biochemical tests were all within normal ranges, and the T-spot test, oncological biomarkers, and purified protein derivative (PPD) test yielded negative outcomes. Chest contrast-enhanced CT revealed a space-occupying lesion at the bronchial opening of the right upper lobe, with uneven enhancement (Figures 1A–C). On the fifth day of hospitalization, flexible bronchoscopy (Figure 1D) and biopsy were performed. However, owing to the challenges linked with performing a biopsy in the right upper lobe, only a few specimens could be obtained. Due to unsatisfactory biopsy results, a multidisciplinary consultation was conducted, and a right superior lobectomy was recommended due to the high possibility of a bronchial tumor being the underlying condition. Eventually, a right upper lobectomy was performed. The lesion was a firm mass with a faint yellow surface. Hematoxylin and eosin (HE) staining of the specimen showed that the tumor cells were arranged in nests or glandular tubes, with abundant cytoplasm and epithelioid appearance, and the area was rich in mucus (Figure 1E). Histopathological examination revealed a PMEC with tumor-free surgical margins and metastasis to the mediastinal lymph node. Immunohistochemical analysis yielded remarkably positive results for the expression of cytokeratin (CK), CK-7, CK-19, and periodic acid-schiff stain (Figure 1F), while showing relatively weaker positivity for epithelial membrane antigen (EMA). Synaptophysin expression was notably absent. No recurrence was observed during the 3-month postoperative follow-up.

### Case 2

**Figure 2:**
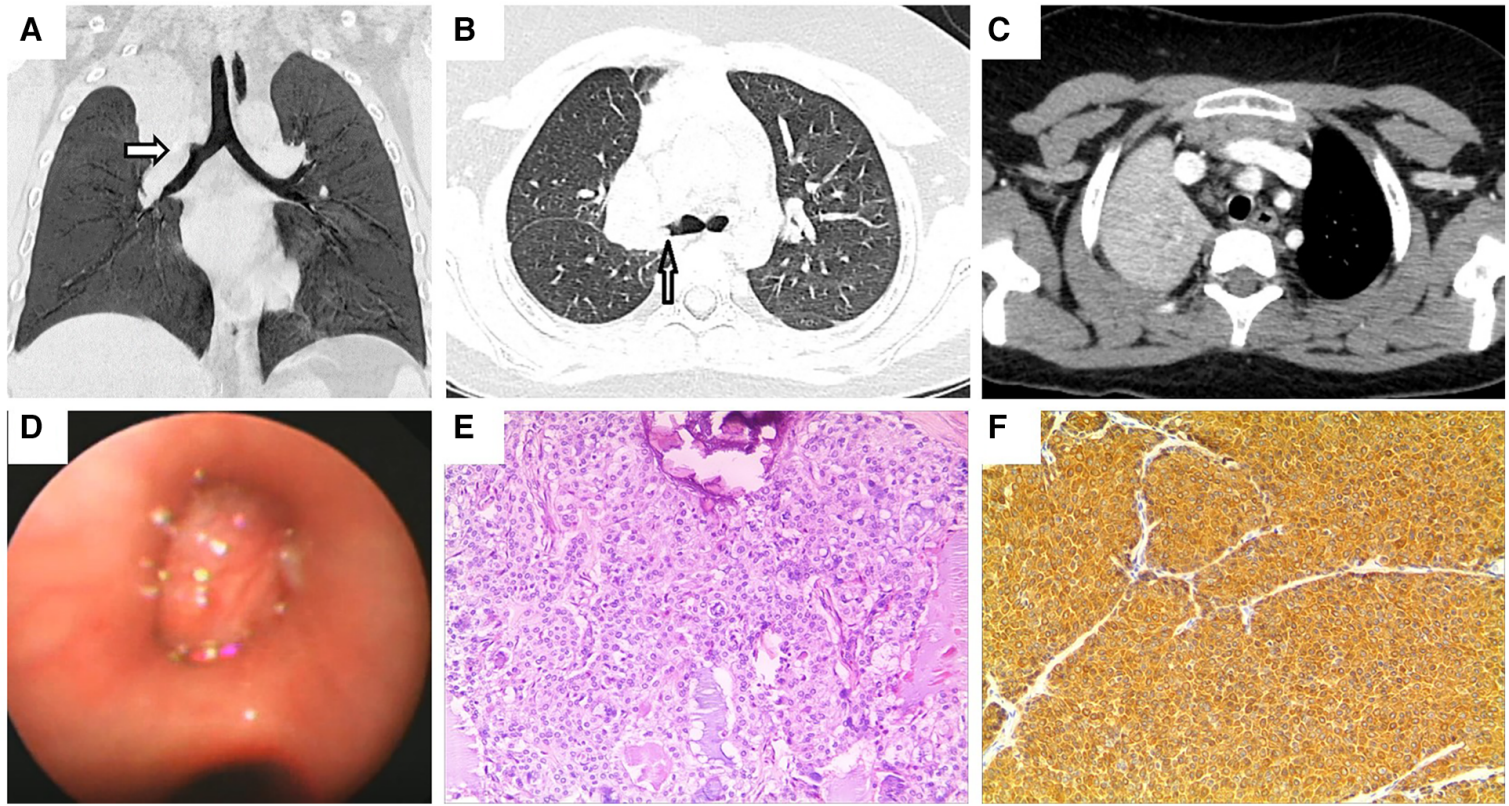
Chest enhanced CT, bronchoscopy, pathological image and immunohistochemistry assay of case 2. Contrast-enhanced CT of the chest shows (**A,B**) a blockage of the right superior lobe bronchus. Arrows indicate space-occupying lesions. (**C**) Atelectasis of the right superior lobe. (**D**) Visual representation of flexible bronchoscopy, confirming the presence of a mass in the right superior lobular bronchus. (**E**) Photomicrograph (HE × 100) shows the confirmed diagnosis of pulmonary mucoepidermoid carcinoma. (**F**) Photomicrograph shows positive immunohistochemical staining for CK (magnification × 100).

A previously healthy 9-year-old boy was admitted to the hospital owing to two instances of hemoptysis within 4 weeks. The volume of expectorated blood was small, appearing as dark red blood clots. Except for a paroxysmal cough before hemoptysis, the patient did not manifest any cough at presentation. He also was devoid of experiencing any fever, shortness of breath, chest tightness, epistaxis, vomiting, or abdominal pain. No abnormalities were detected by physical examinations. Blood routine as well as biochemical tests yielded results that were within normal ranges. Similarly, assessments such as the T-spot test, oncological biomarkers, and PPD test yielded negative results. Chest enhanced CT revealed the presence of a mass in the right upper lobe, invading the right upper lobe bronchus, causing occlusion of the upper lobe bronchus. This radiological finding raised suspicions of malignancy, supported by concurrent mediastinal lymph node metastasis and atelectasis of the right upper lobe (Figures 2A–C). Flexible bronchoscopy (Figure 2D) and biopsy revealed a mucoepidermoid carcinoma in the right superior lobe bronchus. The patient subsequently received a thoracoscopic right upper lobectomy and dissection of the hilar lymph node. HE staining of the excised tissue specimens showed a variety of mucoid cells, epidermoid cells, and intermediate cells, some of which were solid and others were arranged in a glandular tube configuration (Figure 2E). Pathological results confirmed the diagnosis of PMEC, further confirming the absence of tumor involvement at the surgical margins and the presence of mediastinal lymph node metastasis. Immunohistochemical analysis yielded remarkably positive results for the expression of CK, CK-7, CK-19 and EMA (Figure 2F), while showing weaker positivity for chromogranin A and CK-20. Synaptophysin expression was absent. No recurrence was observed during the postoperative follow-up period of 3.5 years.

## Discussion

Primary neoplasms originating in the tracheobronchial tree and lungs are rare, with the majority being malignant. PMEC tumor, is hallmarked by a combination of squamous, mucus-secreting, and intermediate cell types, and is defined by the World Health Organization (2). The incidence of PMEC constitutes approximately 0.1%–0.2% of all primary lung cancers (3). Although PMEC can manifest across all age groups (range, 3–78 years), it is more common in individuals aged 30–50 years (4). A study conducted in Taiwan has confirmed a higher incidence in males than in females (5), whereas other reports have demonstrated an equal distribution between the two genders (6, 7). In our report, we presented two cases, involving one male and one female patient, each. The incidence of PMEC in children is rare, with approximately 55 cases only reported currently (1). In the past 5 years, only two cases have been diagnosed in our hospital, both of which occurred in 9-year-old patients.

PMEC typically presents as an exophytic intrabronchial mass that can have intact or ulcerated bronchial mucosa. The tumors are situated in the submucosal layer of the larger bronchi under microscopy (8). When the tumor is small, patients might not experience typical symptoms. However, as the tumor gradually increases in size, symptoms such as cough, expectoration, fever, and hemoptysis may manifest. In more advanced cases, obstruction of the bronchial lumen can lead to additional changes. Therefore, chest CT and flexible bronchoscopy should be promptly performed when patients present with recurrent cough, sputum, hemoptysis, or atelectasis. These tumors usually originate in the bronchial mucous glands located in the main bronchial trunk or the proximal bronchus of a lobe and are less frequently found in the segmental bronchus and trachea. The growth pattern involves polypoid formations in the bronchus, covered by normal respiratory epithelium (1, 9). Therefore, bronchial lavage and brushing are rarely used for diagnosis, and forceps biopsy is a necessary approach (10). In our study, all the observed lesions were located in the right upper lobe bronchi.

According to classification, PMEC can be low or high grade, depending on nuclear pleomorphism, mitotic activity, and the presence or absence of necrosis. Generally, low-grade tumors are more common in children (1, 3, 4, 9, 11–16). Until the year 2000, only 54 cases of PMEC in children had been reported, of which 92.5% (50/54) were low-grade cancers (9, 15). Conversely, high-grade tumors are more common in adults (5, 17–19). Hsieh et al. (5) reported 41cases in adults, among which 10 patients (24.4%) had low-grade tumors, whereas, the rest (75.6%) had high-grade tumors. Among the 41 patients, 22 patients were older, with only one patient developing low-grade tumor; whereas the rest 21 older patients were all diagnosed with high-grade tumors. Jiang et al. (18) confirmed that 25 of the 34 (73.5%) adult patients had low-grade tumors.

Fluoro-18-fluoro-deoxyglucose positron emission tomography/computed tomography (18F-FDG PET/CT) combines anatomical position location and morphological date from CT with functional data availed by PET, significantly influencing lung cancer diagnosis and staging (20). The use of PET was first reported in a 5-year-old girl with low-grade PMEC (21). The maximum standardized uptake value (SUVmax) is a common semi-quantitative parameter that shows tumor glucose metabolism and estimates tumor proliferation on PET and CT scans. Park et al. (22) employed 18F-FDG PET/CT to predict PMEC individuals’ prognosis and the pathological grade. The authors affirmed that patients with SUVmax higher than 6.5 were predisposed to lymph node metastasis, high-grade PMECs, and recurrences, based on an analysis of 23 patients. Jindal et al. (23) showed that SUVmax values on 18F-FDG PET-CT scans ranged from 0 to 6.2 and 2.86 to 23.4 in low-grade and high-grade PMEC cases, respectively. The authors suggested that PET/CT might play a role in tumor differentiation by predicting the histopathological prognosis. However, these two studies mainly involved adults, and only rare cases of 18F-FDG PET/CT use in children with PMEC have been reported. The comprehensive 18F-FDG PET imaging findings for PMEC in children have not been well established as per our knowledge. Low-grade PMEC was reported in a 15-year-old girl and an 11-year-old boy, yielding SUVmax values of 6.2 and 3.8, respectively (24, 25).

PMEC exhibits diverse immunohistochemical staining patterns, and no single stain can be definitively attributed to its pathogenesis. Tumors showed positive staining for markers such as p40, CK 5/6, and p63. In addition, staining with a keratin cocktail, CK 7, Muc5AC, and CEA may also yield positive results (26). In a study conducted by Andy et al. (27) involving 25 patients with PMEC, all patients showed expression of p63. Hu et al. (28) reported that the positive percentages for p63, CK7, Muc5AC, p40, and CK5/6 were 100%, 100%, 100%, 96.3%, and 50%, respectively. Human epidermal growth factor receptor 2, Napsin A and TTF-1, were all negative (27, 28). In our two patients, both CK and CK7 were determined to be positive.

The most common genetic change in PMEC is the *t*(11; 19) (q21; p13) translocation, which culminates in the generation of the fusion protein mucoepidermoid carcinoma translocated 1–mammalian mastermind like 2 (*MECT::MAML2*) genes (3, 28, 29). This genetic change involves the fusion of exon 1 of a novel gene on chromosome 19, mucoepidermoid carcinoma translocated 1 (*MECT1*), with exons 2–5 of a gene located on chromosome 11 that is linked to the Notch signaling pathway, known as mastermind-like 2 (*MAML2*) (30). Achcar et al. (19) showed *MAML2* rearrangement in 13 out of 17 (77%) cases of PMEC. Subsequent research has indicated that the *MECT::MAML2* fusion product is specific to PMEC and associated with a subset of tumors that exhibit a more favorable prognosis owing to their extended clinical course (31). In our study, the detection of *MECT::MAML2* was not feasible owing to limitations in specimen collection. The precise role of this reciprocal translocation and resultant fusion protein in the development of PMEC warrants further investigation in a large number of patients, especially among children.

Surgical resection is the recommended approach for PMEC, with an emphasis on achieving complete excision through lobectomy, sleeve resection, or other surgical methods based on the location of the tumor (8, 18, 32, 33). In low-grade PMEC cases, efforts are made to minimize the removal of normal lung tissue. Prognostic factors that predict unfavorable survival encompass the histological tumor grade, TNM stage, patient age, and the extent of resection (5, 34, 35). Jiang et al. pointed out that lymph node metastasis was the sole independent prognostic factor (18).

In children, PMEC tends to show low-grade malignancy, and the long-term prognosis after surgical resection is excellent (1, 3, 9, 11, 13, 16, 36–38). Granata et al. (3) confirmed that among 51 pediatric patients, 49 patients with low-grade tumors experienced no tumor recurrence or metastasis after surgery (mean follow-up, 5 years and 3 months; range, 8 months to 23 years). Among the remaining two patients with high-grade tumors, one was lost to follow-up, whereas the other remained disease-free after a 6-year follow-up. A study involving 34 adults with PMEC revealed that 7 patients experienced tumor recurrence or metastasis, including 4 who died (mean follow-up period, 63 months). All patients’ 5-year overall survival and progression-free survival rates, were 84.6% and 81.6%, correspondingly. In our study, neither of the two patients experienced recurrence, and the longest follow-up period was 3.5 years. Overall, children with PMEC show a better prognosis compared to adults.

In conclusion, PMEC is a rare malignant neoplasm, especially in children. When patients present with repeated cough, sputum production, hemoptysis, or atelectasis, chest CT and flexible bronchoscopy should be performed promptly to facilitate timely diagnosis and consideration of PMEC. Surgical resection is an effective treatment strategy for managing patients with PMEC. However, further studies are warranted to better understand PMEC, particularly in children.

## Data Availability

The original contributions presented in the study are included in the article/Supplementary Material, further inquiries can be directed to the corresponding authors.
